# The effect of denture-wearing on physical activity is associated with cognitive impairment in the elderly: A cross-sectional study based on the CHARLS database

**DOI:** 10.3389/fnins.2022.925398

**Published:** 2022-08-16

**Authors:** Yisheng Chen, Zhiwen Luo, Yaying Sun, Yifan Zhou, Zhihua Han, Xiaojie Yang, Xueran Kang, Jinrong Lin, Beijie Qi, Wei-Wei Lin, Haoran Guo, Chenyang Guo, Ken Go, Chenyu Sun, Xiubin Li, Jiwu Chen, Shiyi Chen

**Affiliations:** ^1^Department of Sports Medicine, Huashan Hospital, Fudan University, Shanghai, China; ^2^Department of Ophthalmology, Shanghai General Hospital, Shanghai Jiao Tong University School of Medicine, Shanghai, China; ^3^Department of Ophthalmology, Putuo People’s Hospital, Tongji University, Shanghai, China; ^4^Department of Orthopedics, Shanghai General Hospital, Shanghai Jiao Tong University School of Medicine, Shanghai Jiao Tong University, Shanghai, China; ^5^Department of Stomatology, Shanghai General Hospital, Shanghai Jiao Tong University School of Medicine, Shanghai Jiao Tong University, Shanghai, China; ^6^Shanghai Jiao Tong University School of Medicine, Shanghai Jiao Tong University, Shanghai, China; ^7^Department of Neurosurgery, Second Affiliated Hospital of Zhejiang University School of Medicine, Zhejiang University, Hangzhou, China; ^8^Chinese PLA Medical School, Beijing, China; ^9^St. Marianna Hospital, Tokyo, Japan; ^10^AMITA Health Saint Joseph Hospital Chicago, Chicago, IL, United States; ^11^Department of Neurology, The Second Affiliated Hospital of Shandong First Medical University, Shanghai, China

**Keywords:** denture, cognitive impairment, physical activity, tooth loss, the China health and retirement longitudinal study (CHARLS), environment, phenotype

## Abstract

**Background:**

Currently, only a few studies have examined the link between dental health, cognitive impairment, and physical activity. The current study examined the relationship between denture use and physical activity in elderly patients with different cognitive abilities.

**Methods:**

The study data was sourced from the 2018 China Health and Retirement Longitudinal Study (CHARLS) database, which included information on denture use and amount of daily physical activity undertaken by older persons. Physical activity was categorized into three levels using the International Physical Activity General Questionnaire and the International Physical Activity Scale (IPAQ) rubric. The relationship between denture use and physical activity in middle-aged and older persons with varying degrees of cognitive functioning was studied using logistic regression models.

**Results:**

A total of 5,892 older people with varying cognitive abilities were included. Denture use was linked to physical activity in the cognitively healthy 60 + age group (*p* = 0.004). Denture use was positively related with moderate physical activity in the population (odds ratio, OR: 1.336, 95% confidence interval: 1.173–1.520, *p* < 0.001), according to a multivariate logistic regression analysis, a finding that was supported by the calibration curve. Furthermore, the moderate physical activity group was more likely to wear dentures than the mild physical activity group among age-adjusted cognitively unimpaired middle-aged and older persons (OR: 1.213, 95% CI: 1.053–1.397, *p* < 0.01). In a fully adjusted logistic regression model, moderate physical activity population had increased ORs of 1.163 (95% CI: 1.008–1.341, *p* < 0.05) of dentures and vigorous physical activity population had not increased ORs of 1.016 (95% CI: 0.853–1.210, *p* > 0.05), compared with mild physical activity population.

**Conclusion:**

This findings revealed that wearing dentures affects physical activity differently in older persons with different cognitive conditions. In cognitively unimpaired older adults, wearing dentures was associated with an active and appropriate physical activity status.

## Introduction

Of late, oral diseases have emerged as a global public health problem. Approximately 55% of the population suffers from varying degrees of oral diseases, with the elderly being particularly at risk; consequently, oral diseases pose a major disease burden (Quine and Morrell, 2009; Peres et al., 2019). Oral diseases can be caused by aging, digestive and endocrine system dysfunction, chronic stress, nutritional deficiencies, and unhealthy lifestyle habits (Jin et al., 2016; Pflipsen and Zenchenko, 2017; Peres et al., 2019). In developing countries, oral diseases are largely treated ineffectively, and the elderly are more likely to be affected by them and have difficulty accessing dental care (Quine and Morrell, 2009; Peres et al., 2019). Oral diseases not only affect the physiological functions of the mouth, such as chewing and pronunciation, but are also closely related to heart disease, stroke, diabetes, and other systemic diseases, thereby endangering the quality of life of the middle-aged and elderly populations (Nazir et al., 2018; Gondivkar et al., 2019). Therefore, oral diseases are closely associated with malnutrition and systemic diseases. These problems can be prevented with dentures (Preshaw et al., 2011; da Mata et al., 2019; Bannwart et al., 2021).

Studies have shown an association between oral health status and cognitive dysfunction, with poor cognitive status often being accompanied by poor oral health status (Delwel et al., 2018; Farsai, 2021). People who do not wear dentures have a reduced masticatory function, which may be associated with mild cognitive impairment, and dentures may improve this condition (Kim et al., 2020). In addition, among patients with Alzheimer’s, those who wore dentures were demonstrated to have a higher mortality rate than those who did not (Yun et al., 2020). Denture wear may be associated with a protective effect on cognitive function in the elderly, based on these studies. However, research on how dentures protect cognitive function in the elderly is lacking. This study aimed to analyze the factors influencing the cognitive status of older adults in China comprehensively and systematically.

The academic community has been paying increasing attention in recent years to systemic frailty caused by oral frailty, and oral health is a useful screening tool for early debilitating symptoms (Watanabe et al., 2020). For instance, the deterioration of oral health induces frailty in patients with cardiovascular disease (Ogawa et al., 2021). Thus, the deterioration of oral health is a risk factor for the development of frailty, and frailty accelerates the onset of cognitive decline in the elderly (Jongsiriyanyong and Limpawattana, 2018). Although dentures can improve oral function, not all patients with tooth loss wear them, a phenomenon that is particularly prevalent in developing countries. In addition to preventing frailty and slowing dementia progression, physical activity, including aerobic exercise, has also been found to prevent frailty (Panza et al., 2018). Interestingly, the willingness of the population to participate in physical activity was found to be positively associated with improvements in oral health (Sanchez et al., 2020). Therefore, we hypothesized that improved oral function (e.g., chewing, speaking, and appearance functions) with dentures could promote the willingness of the population to be physically active, which could potentially explain the prevention of cognitive impairment and related systemic diseases in the elderly with dentures.

Much has been written about the mutual promotion of physical activity and physical health through the ages (De la Rosa et al., 2020). However, the relationship between denture-related oral health and physical activity remains unclear. The China Health and Retirement Longitudinal Study (CHARLS) contains high-quality microdata representing households and individuals aged 45 and older in China, which allows researchers to analyze population aging and promote interdisciplinary research (Strauss et al., 2010; Zhao et al., 2021; Zhou et al., 2022). The purpose of this study is to investigate the relationship between denture-wearing and physical activity in older adults with different cognitive function states, in order to provide new data and reference points to slow down cognitive decline and improve seniors’ quality of life.

## Methods

### Study sample

**FIGURE 1:**
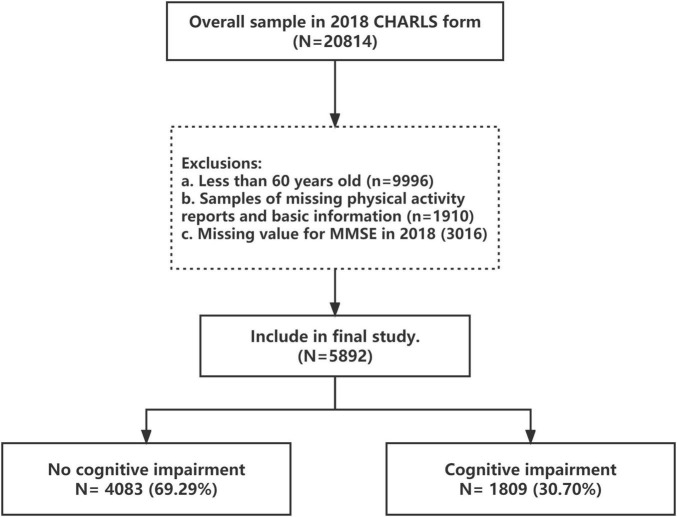
Study flow.

Data was sourced from the 2018 CHARLS database. This database is a national survey study among Chinese adults aged ≥45 years, and uses multi-stage sampling and probability-proportionate-to-size sampling at the urban and rural areas administrative unit sampling stages (Strauss et al., 2010; Zhong, 2011). The CHARLS study sample consists of 150 district-level units (scattered across 28 provincial units nationwide), 450 rural areas-level units, and covers a total of 20,814 individuals. The survey method uses face-to-face interviews to provide detailed information on demographics, health status, socioeconomic status, and lifestyle habits. It makes it possible to estimate the population’s oral health and analyze the association between physical activity levels and cognitive impairment in old age. The sample used in this study was 10818 elderly people aged 60 years and older who had been surveyed as part of CHARLS in 2018, excluding 322 cases with missing age values, 1910 cases with missing physical activity information, and 3016 cases with missing cognitive function information. Finally, the number of eligible samples amounted to 5892 cases, comprising 3,162 men and 2,730 women (Figure 1).

### Physical activity judgment criteria

Participants were randomly selected to answer questions about their physical activity. The International Physical Activity General Questionnaire categorizes physical activity intensity into three categories: vigorous physical activity, moderate physical activity, and mild physical activity. Vigorous physical activity includes physically demanding activities similar to heavy lifting, farming, aerobics, and digging. Moderate physical activity includes bicycling at regular speed, mopping up, tai chi, carrying light things, walking at a brisk pace, and so on. Mild physical activity includes walking for work, as well as activities for recreation, exercise, or leisure purposes. The intensity of physical activity, the time spent on activity per week, and the duration of activity per day are all included in each physical activity assessment. The duration of each activity is categorized into four levels: <0.5 h, ≥0.5 h but less than 2 h, ≥2 h but less than 4 h, and ≥ 4 h. Further, the physical activity level of the elderly is classified into low, medium, and high intensity according to the International Physical Activity Scale (IPAS) (Garber et al., 2011).

### Determination of cognitive impairment

Cognitive impairment was evaluated using the Mini-Mental State Examination scale (MMSE), which has a total score of 30 points (Folstein et al., 1975). The MMSE includes questions on memory, orientation, attention and numeracy, recall, as well as language skills. As described in previous studies, cognitive impairment was defined as MMSE scores < 17, < 20, and < 24 for illiterate individuals, those who had studied up to elementary school, and those who had studied up to secondary school, respectively (Chen et al., 2021).

### Chronic disease assessment

Based on the answer to the question “Have you been diagnosed by a doctor as XX?” it was determined whether the patient had hypertension; dyslipidemia; diabetes; chronic lung diseases; heart attack; stroke; kidney disease; memory related disease; brain damage; emotional, nervous or psychiatric problems; cancer or malignant tumor; asthma; arthritis or rheumatism; liver disease, stomach, or other digestive diseases. These chronic conditions were categorized as “yes” or “no” and were selected based on a longitudinal pilot study. Self-reported health status was also recorded with the question “How satisfied are you with your health status?” The answers ranged across “very poor,” “poor,” “fair,” “good,” and “very good.”

### Demographic variables and personal health-related behaviors

Demographic variables included age, sex, education, marital status, and region of residence. Educational levels included illiteracy elementary-school education, and secondary school education. Marital status was categorized as divorced or separated, married, never married, and widowed. The region of residence included the rural and urban areas. Personal health-related behaviors included smoking, alcohol consumption, daily sleep duration, and frequency of social activities. Smoking status was c classified into never smoked, formerly smoked but quit, and currently smoking. The frequency of alcohol consumption was reported as more than once a month, less than or equal to once a month, and never. Patients’ nap times and total sleep time during the day were collected, which allowed an inference of patients’ nighttime sleep duration. In addition, participants were considered to have a history of falls if they answered that they had a fall in the past two years.

### Assessment of life quality

Activities of daily living (ADL) and Instrumental Activities of Daily Living (IADL) were used as indicators to evaluate the status of basic life activities and instrumental life activities, respectively. The six ADL items were dressing, bathing, eating, transferring, self-control, and toileting (Lawton and Brody, 1969; Gill et al., 1995). The six IADL items were doing housework, cooking, shopping, making phone calls, taking medication, and managing money. As described in a previous study, for each ADL or IADL task, functional ability was measured on a four-point scale (Zhang et al., 2021). We summed the scores on the ADL and IADL tasks as the ADL and IADL scores, respectively. For the disability data, participants were also asked whether they had physical disabilities, vision problems, hearing problems, or speech impediments. Answers were categorized as either “yes” or “no”.

### Statistical analyses

Statistical analyses and graphical representations were performed using R software, version 4.0.2 (The R Foundation for Statistical Computing, Vienna, Austria) and SPSSAU (Version 22.0) [Online Application Software], a web tool^1^. Descriptive statistics were analyzed for basic demographic characteristics, somatic health, and health behaviors of the study participants with different cognitive statuses. For normally distributed data, the means and standard deviations (SDs) are described; for non-normally distributed variables, the medians and interquartile ranges (IQRs). The *t*-test was used to analyze differences between normally distributed, quantitative variables. The Wilcoxon test was used to compare differences between non-normally distributed variables, and a chi-square test was used for comparisons between groups. Multifactorial analysis was performed using logistic regression models to clarify the effects of different factors on the physical activity of older adults as previous researches (Chen et al., 2020, 2022; Dong et al., 2021; Shi et al., 2021; Ying et al., 2021; Mo et al., 2022). GiViTI method can be used both to evaluate the internal calibration (i.e., the goodness of fit) and to assess the validity of an externally developed model. As shown in previous studies, the function of logistic regression models was evaluated using the GiViTI method, and the RMS-package was used to plot the GiViTI calibration belt (Poole et al., 2012; Jiang et al., 2022). Significance level of α was set at 0.05. Effect sizes are shown in the form of odds ratios (OR) and their corresponding 95% confidence intervals (95% CI).

## Results

### Characteristics of samples

Among the 5,892 cases investigated, 4,083 (69.30%) had no cognitive impairment, including 2,426 (59.41%) without dentures and 1,657 (40.58%) with dentures. A total of 1,809 cases (30.70%) had cognitive impairment, including 1,185 (65.50%) without dentures and 624 (35.50%) with dentures. Supplementary Table 1 shows the characteristics of the included participants. For those with no cognitive impairment, the age, sex, place of residence, marital status, social activity, physical disabilities, physical activity, and MMSE scores were significantly different between the groups with and without dentures. For the population with cognitive impairment, only sex, presence of dyslipidemia, history of heart attack, and MMSE scores were statistically significantly different (Supplementary Table 1). MMSE values were higher in those wearing dentures including those with and without cognitive impairment, suggesting that cognitive levels indicated by MMSE scores were higher in older patients wearing dentures than in those without dentures (*p* < 0.05). However, wearing dentures was found to be associated with physical activity only in those who did not have cognitive impairment (*p* = 0.004).

### Logistic regression analysis to determine the factors associated with denture use in the presence or absence of cognitive impairment

**FIGURE 2:**
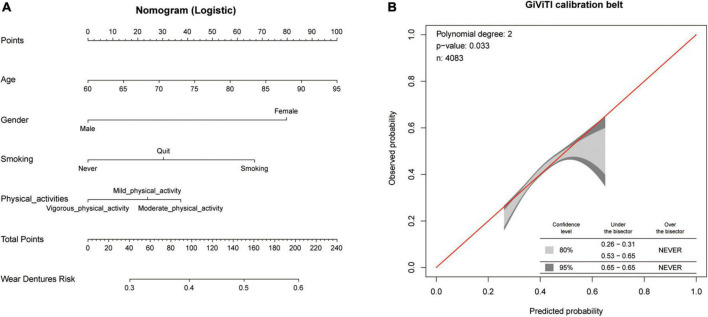
Logistic regression analysis to determine the factors associated with denture use in the presence or absence of cognitive impairment. **(A)** Denture wearing status was predicted by the nomogram prediction model based on age, sex, smoking status, and physical activity. A nomogram consists of three main parts: (I). the scoring part, which represents each variable at different values by a single score; (II). the indicator part, which includes such variables as age, sex, smoking, and physical activity. On the line segment, each indicator is marked with a scale that indicates its range of values. Indicators contribute to the ending event based on their length of line segments in the diagram; (III). the ending segment, i.e., total points and wear dentures risk in the diagram, where total points indicate the total score of all indicators after taking the values of the corresponding individual scores together. Probability indicates the likelihood of achieving a goal when an event occurs; **(B)** This nomogram’s calibration plots.

As shown in Table 1, the relationships between sociodemographic features, social relationships, personal health information, and denture use in this study were included in the one-way logistic regression model for analysis. For those with no cognitive impairment, advanced age, being male, smoking, social activity, dyslipidemia, and physical activity were found to be associated with denture use in the univariate regression analysis (*p* < 0.05). Meanwhile, age (OR = 1.032, 95% CI: 1.020–1.044, *p* < 0.001), sex (OR = 0.603, 95% CI: 0.500–0.727, *p* < 0.001), smoking status (OR = 0.786, 95% CI: 0.653–0.947, *p* = 0.011), and physical activity (OR = 1.336, 95% CI: 1.173–1.520, *p* < 0.001) were found to be associated with denture use in the multivariate logistic regression analysis. For the population with cognitive impairment, sex, smoking, asthma, and liver disease were found to be associated with denture use on univariate regression analysis (*p* < 0.05). Among these, only asthma (OR = 0.587, 95% CI: 0.357–0.963, *p* = 0.035) was found to be associated with denture use in the multivariate logistic regression analysis. To further visualize the ability of age, sex, smoking status, and physical activity to predict denture use, a nomogram prediction model was plotted (Figure 2A). Denture wearing status was predicted by the nomogram prediction model based on age, sex, smoking status, and physical activity. The GiViTI calibration belt suggested that the model had a fair predictive power ranging from 30 to 60% (Figure 2B). The above findings suggested that denture use was positively associated with moderate physical activity in people without cognitive impairment. Accordingly, denture-wearing may promote moderate physical activity in the non-cognitively impaired population, but this positive effect was masked by cognitive impairment in the cognitively impaired population.

**TABLE 1:** Univariate and multivariable logistic regression analysis of patients with and without dentures.

Variables	No cognitive impairment				Cognitive impairment			

	**Univariate analysis**		**Multivariate analysis**		**Univariate analysis**		**Multivariate analysis**	
		
	OR (95% CI)	*P*-value	OR (95% CI)	*P*-value	OR (95% CI)	*P*-value	OR (95% CI)	*P*-value
Age	1.027 (1.016-1.039)	< 0.001	1.032 (1.020-1.044)	< 0.001**	1.013 (0.998-1.028)	0.091	1.022 (0.999- 1.045)	0.056
Sex	0.713 (0.629-0.810)	< 0.001	0.603 (0.500-0.727)	< 0.001**	0.714 (0.586-0.870)	< 0.001	0.773 (0.526- 1.136)	0.191
Marital_status	0.946 (0.553-1.617)	0.838	NA		0.424 (0.182-0.989)	0.047	0.400 (0.130- 1.230)	0.11
Smoking	1.146 (1.008-1.303)	0.038	0.786 (0.653-0.947)	0.011*	1.440 (1.173-1.769)	< 0.001	NA	
Drinking	0.822 (0.648-1.042)	0.106	NA		0.667 (0.431-1.030)	0.068	0.453 (0.143- 1.439)	0.179
Social_activity	1.353 (1.191-1.536)	< 0.001	NA		1.133 (0.933-1.377)	0.207	NA	
Dyslipidemia	0.850 (0.722-1.000)	0.05	NA		0.877 (0.650-1.185)	0.393	NA	
Asthma	0.875 (0.645-1.186)	0.389	NA		1.594 (1.002-2.536)	0.049	0.587 (0.357- 0.963)	0.035*
Liver_disease	1.087 (0.799-1.479)	0.596	NA		0.588 (0.365-0.947)	0.029	0.715 (0.450- 1.138)	0.157
Physical_activities	1.173 (1.019-1.349)	0.026	1.336 (1.173-1.520)	< 0.001**	1.111 (0.881-1.402)	0.373	NA	

All analyses were conducted at a 5% significance level; CI confidence interval; * p < 0.05, ** p < 0.01.

^a^ The multivariate model of patients without cognitive impairment incorporated four predictors, including age, gender, smoking, and physical activities.

^b^ The final model of patients with cognitive impairment incorporated six predictors, including age, gender, marital status, drinking, asthma, and liver disease.

### Characteristics of study participants without cognitive impairment

**FIGURE 3:**
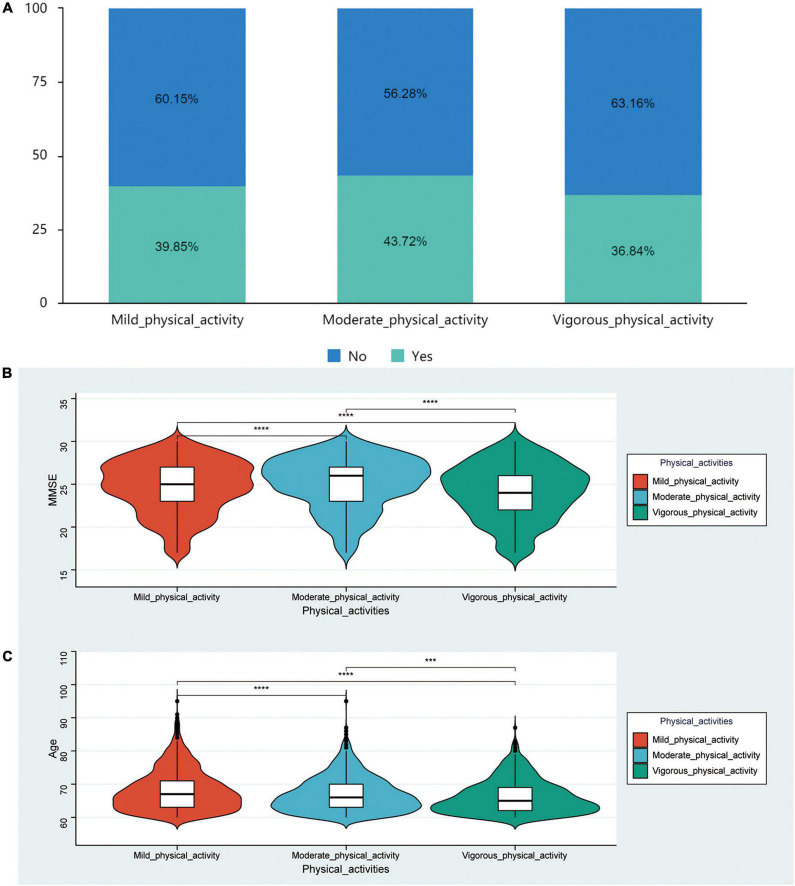
Analysis of factors influencing physical activity. **(A)** Bar graph showing the difference in the proportion of denture wearing in different physical activities. **(B,C)** Bar graph showing the difference in MMSE**(B)** and Age **(C)** in different physical activities. ***p < 0.001, ****p < 0.0001.

Among the 4,083 patients without cognitive impairment, 809 performed vigorous physical activity, 1,402 performed moderate physical activity, and 1,872 performed mild physical activity. Among the different physical activity-groups, age, sex, denture use, residence, education, marital status, drinking, social activity, self-report health conditions, cognitive impairment, comorbidities (chronic lung diseases, history of heart attack, history of stroke, cancer or malignant tumor), physical disabilities, ADL, and MMSE scores were significantly different (*p* < 0.05) (Table 2). This suggested that the intensity of physical activity in the population was significantly associated with their cognitive level, physical health status, basic demographic characteristics, and the quality of life. In the present study, we also found that the proportion of denture use in the non-cognitively impaired population was the highest in the moderate physical activity population (*p* = 0.004) (Figure 3A). The mean MMSE score was higher in the moderate physical activity group relative to the others (*p* < 0.001) (Figure 3B). The mean age of the mild physical activity population was greater than that of the moderate physical activity and vigorous physical activity populations (Figure 3C). This suggested that the physical activity level of the population decreased with age.

**TABLE 2:** Baseline characteristics of study participants without cognitive impairment according to physical activity status.

Characteristics	Physical activities(%)			Total	*P*-value
	Mild physical activity (*n* = 1872)	Moderate physical activity (*n* = 1402)	Vigorous physical activity (*n* = 809)		
Age	67 (63,71)	66 (63,70)	65 (62,69)		< 0.001***
**Sex**					
Female	782 (41.77)	699 (49.86)	236 (29.17)	1,717 (42.05)	< 0.001***
Male	1,090 (58.23)	703 (50.14)	573 (70.83)	2,366 (57.95)	
**Wear dentures**					
No	1126 (60.15)	789 (56.28)	511 (63.16)	2,426 (59.42)	0.004**
Yes	746 (39.85)	613 (43.72)	298 (36.84)	1,657 (40.58)	
**Residence**					
Urban	761 (40.89)	598 (42.93)	115 (14.30)	1,474 (36.32)	< 0.001***
Rural areas	1,100 (59.11)	795 (57.07)	689 (85.70)	2,584 (63.68)	
**Education**					
Elementary	929 (49.63)	629 (44.86)	430 (53.15)	1,988 (48.69)	< 0.001***
Illiteracy	255 (13.62)	194 (13.84)	155 (19.16)	604 (14.79)	
Secondary	688 (36.75)	579 (41.30)	224 (27.69)	1,491 (36.52)	
**Marital status**					
Divorced or Separated	25 (1.34)	22 (1.57)	9 (1.11)	56 (1.37)	< 0.001***
Married	1,582 (84.51)	1180 (84.17)	742 (91.72)	3,504 (85.82)	
Never married	10 (0.53)	4 (0.29)	2 (0.25)	16 (0.39)	
Widowed	255 (13.62)	196 (13.98)	56 (6.92)	507 (12.42)	
**Smoking**					
Never	908 (90.62)	772 (91.15)	326 (90.56)	2,006 (90.81)	0.542
Quit	45 (4.49)	39 (4.60)	22 (6.11)	106 (4.80)	
Smoking	49 (4.89)	36 (4.25)	12 (3.33)	97 (4.39)	
**Drinking**					
Drink but less than once a month	141 (7.53)	136 (9.70)	80 (9.89)	357 (8.74)	< 0.001***
Drink more than once a month	508 (27.14)	400 (28.53)	331 (40.91)	1,239 (30.35)	
No	1,223 (65.33)	866 (61.77)	398 (49.20)	2,487 (60.91)	
**Social activity**					
No	886 (47.33)	476 (33.95)	391 (48.33)	1,753 (42.93)	< 0.001***
Yes	986 (52.67)	926 (66.05)	418 (51.67)	2,330 (57.07)	
**Self-report health conditions**					
Fair	923 (49.31)	749 (53.42)	426 (52.66)	2,098 (51.38)	< 0.001***
Good	206 (11.00)	185 (13.20)	108 (13.35)	499 (12.22)	
Poor	430 (22.97)	253 (18.05)	146 (18.05)	829 (20.30)	
Very good	193 (10.31)	158 (11.27)	95 (11.74)	446 (10.92)	
Very poor	120 (6.41)	57 (4.07)	34 (4.20)	211 (5.17)	
**Hypertension**					
No	981 (81.95)	746 (82.71)	500 (84.75)	2,227 (82.82)	0.337
Yes	216 (18.05)	156 (17.29)	90 (15.25)	462 (17.18)	
**Dyslipidemia**					
No	1,275 (86.27)	968 (85.36)	638 (88.37)	2,881 (86.41)	0.179
Yes	203 (13.73)	166 (14.64)	84 (11.63)	453 (13.59)	
**Diabetes**					
No	1,519 (93.94)	1162 (92.74)	717 (94.72)	3,398 (93.69)	0.179
Yes	98 (6.06)	91 (7.26)	40 (5.28)	229 (6.31)	
**Chronic lung diseases**					
No	1,516 (92.55)	1205 (94.88)	674 (93.48)	3395 (93.55)	0.040*
Yes	122 (7.45)	65 (5.12)	47 (6.52)	234 (6.45)	
**History of heart attack**					
No	1316 (88.92)	1009 (88.66)	675 (93.75)	3,000 (89.87)	0.001**
Yes	164 (11.08)	129 (11.34)	45 (6.25)	338 (10.13)	
**History of Stroke**					
No	1,673 (92.69)	1296 (93.91)	773 (96.87)	3,742 (93.95)	< 0.001***
Yes	132 (7.31)	84 (6.09)	25 (3.13)	241 (6.05)	
**Kidney disease**					
No	1,636 (95.06)	1236 (95.37)	705 (96.58)	3,577 (95.46)	0.252
Yes	85 (4.94)	60 (4.63)	25 (3.42)	170 (4.54)	
**Memory related disease**					
No	1793 (97.71)	1357 (98.62)	787 (98.25)	3,937 (98.13)	0.164
Yes	42 (2.29)	19 (1.38)	14 (1.75)	75 (1.87)	
**Brain damage**					
No	1723 (97.07)	1302 (97.97)	746 (97.64)	3,771 (97.49)	0.273
Yes	52 (2.93)	27 (2.03)	18 (2.36)	97 (2.51)	
**Emotiol nervous or psychiatric problems**					
No	1,826 (98.76)	1375 (99.28)	796 (99.25)	3,997 (99.03)	0.253
Yes	23 (1.24)	10 (0.72)	6 (0.75)	39 (0.97)	
**Cancer or malignant tumor**					
No	1,817 (98.16)	1362 (98.13)	802 (99.50)	3,981 (98.42)	0.022*
Yes	34 (1.84)	26 (1.87)	4 (0.50)	64 (1.58)	
**Asthma**					
No	1,716 (97.06)	1330 (97.87)	765 (98.20)	3,811 (97.57)	0.152
Yes	52 (2.94)	29 (2.13)	14 (1.80)	95 (2.43)	
Arthritis or rheumatism					
No	1,140 (89.83)	835 (88.45)	456 (88.72)	2,431 (89.15)	0.552
Yes	129 (10.17)	109 (11.55)	58 (11.28)	296 (10.85)	
**Liver disease**					
No	1,716 (96.19)	1281 (95.03)	740 (95.85)	3,737 (95.72)	0.278
Yes	68 (3.81)	67 (4.97)	32 (4.15)	167 (4.28)	
**Stomach or other digestive diseases**					
No	1,325 (89.89)	981 (89.75)	554 (90.38)	2,860 (89.94)	0.917
Yes	149 (10.11)	112 (10.25)	59 (9.62)	320 (10.06)	
**Physical disabilities**					
No	1,681 (96.39)	1305 (98.64)	738 (97.36)	3,724 (97.36)	0.001**
Yes	63 (3.61)	18 (1.36)	20 (2.64)	101 (2.64)	
**Vision problem**					
No	1,617 (95.96)	1240 (96.88)	696 (95.47)	3,553 (96.18)	0.236
Yes	68 (4.04)	40 (3.13)	33 (4.53)	141 (3.82)	
**Hearing problem**					
No	1,489 (93.65)	1160 (94.62)	649 (93.65)	3,298 (93.99)	0.516
Yes	101 (6.35)	66 (5.38)	44 (6.35)	211 (6.01)	
**Speech impediment**					
No	1,858 (99.79)	1395 (99.64)	804 (99.88)	4,057 (99.75)	0.531
Yes	4 (0.21)	5 (0.36)	1 (0.12)	10 (0.25)	
**Fallen last two years**					
No	1,519 (81.14)	1140 (81.31)	643 (79.48)	3,302 (80.87)	0.528
Yes	353 (18.86)	262 (18.69)	166 (20.52)	781 (19.13)	
ADL	6 (6,6)	6 (0,6)	6 (0,6)		< 0.001***
IADL	6 (6,7)	6 (6,6)	6 (6,6)		1
MMSE	25 (23,27)	26 (23,27)	24 (22,26)		< 0.001***

*:P < 0.05; **: P < 0.01; ***: P < 0.001.

ADL, activities of daily living; IADL, Instrumental Activities of Daily Living; MMSE, The Mini-Mental State Examination; SE, standard error.

### Multivariate logistic regression models found an association between denture use and moderate physical activity in older adults without cognitive impairment

Table 3 shows that moderate physical activity was 1.173 times more likely to use dentures than those with mild physical activity (95% CI: 1.019–1.349). Both the age-adjusted OR (95% CI) and the age- and sex-adjusted OR (95% CI) showed a higher rate of denture use among those with moderate physical activity (OR = 1.213, 95% CI: 1.053–1.397, *p* < 0.01; OR = 1.184, 95% CI: 1.027–1.365, *p* < 0.05, respectively). A multivariate model adjusting age, sex, and MMSE also showed a higher rate of denture use among those performing moderate physical activity (OR = 1.163, 95% CI: 1.008–1.341, *p* < 0.05). In contrast, the likelihood of denture use was not increased in the vigorous physical activity group (OR = 1.016, 95% CI: 0.853–1.210, *p* > 0.05). Our findings suggested that denture use was associated with active and appropriate physical activity status in cognitively normal middle-aged and older adults.

**TABLE 3:** Association between wear dentures status and physical activity by multivariate logistic regression models in older adults without cognitive impairment.

Variables	Mild physical activity (*n* = 1872)	Vigorous physical activity (*n* = 809)	Moderate physical activity (*n* = 1402)
Total number of people wearing dentures,%	574 (48.44)	310 (26.16)	301 (25.40)
Unadjusted OR (95% CI)	1.00 (ref)	0.880 (0.742-1.044)	1.173 (1.019-1.349)*
Age-adjusted OR (95%CI)	1.00 (ref)	0.929 (0.783-1.104)	1.213 (1.053-1.397)**
Age, Gender-adjusted OR (95%CI)	1.00 (ref)	0.976 (0.820-1.161)	1.184 (1.027-1.365)*
Multivariate-adjusted^a^ OR (95%CI)	1.00 (ref)	1.016 (0.853-1.210)	1.163 (1.008-1.341)*

^a^ Adjusted for age, gender and MMSE; * p < 0.05, ** p < 0.01.

## Discussion

In this study, the interrelationship between denture use and physical activity in the elderly population was investigated using the presence or absence of dentures and physical activity as exposure factors, respectively. As shown in Supplementary Table 1, denture use was positively correlated with age. Interestingly, in the analysis illustrated in Table 2, we found that denture use was associated with more moderate physical activity. However, we also found that the level of physical activity decreased with age in the population. Normally, we assume that those who wear dentures tend to be older, are physically inactive, and exercise lesser. However, a surprising finding that caught our attention was that the physical activity generally increased after wearing dentures. Therefore, we proposed a presumed that denture use would impact physical activity and that this effect will be more pronounced after age correction. We used multivariate logistic regression models to analyze the correlation between denture use and physical activity in older adults without cognitive impairment. After adjusting for confounding factors such as sociodemographic characteristics and cognitive status, we found a correlation between denture use and physical activity in the middle-aged and elderly population. In addition, the GiViTI calibration belt validated the predictive power of the model in this study. Therefore, older adults with cognitively normal physical activity levels use dentures more frequently.

Previous studies suggested that people wearing dentures tended to be older and in poorer health (Castrejon-Perez and Borges-Yañez, 2012; Lee and Sabbah, 2018; Albani et al., 2021). A decline in MMSE scores is a sign of cognitive impairment in old age (Tahami Monfared et al., 2022). We found that those wearing dentures had lower cognitive levels and were older relative to those who did not. Physical inactivity is generally associated with decreased MMSE scores and dementia (López-Ortiz et al., 2021). However, these denture-wearing populations were more likely to have appropriate physical activity. Therefore, wearing dentures may have prevented a decline in physical activity due to declining cognitive levels and aging.

In the middle-aged and older populations, oral disease is usually associated with lifestyle conditions. Moreover, oral disease often leads to changes in oral habits and dietary choices, including poor nutrition, dietary restrictions, alcohol use, and other factors that may increase the risk of frailty or reduce physical activity (Farmer et al., 1988; Albani et al., 2021). Our findings suggest that people who wear dentures, even if they are older and in poorer physical condition, may have sufficient energy to participate in physical activity because of better nutrient intake compared to those with poor oral health who were not properly treated (Gupta et al., 2019). The restoration of oral function has a positive effect on nutritional intake, the prevention of frailty, the prevention of aggravation of cognitive impairment, and the maintenance of mental health (Hakeem et al., 2019; Watanabe et al., 2020). Recovery of all these indicators improves the patient’s ability to care for themselves (Gupta et al., 2019; Sabbah et al., 2020). In the present study, denture use was associated with an active and appropriate physical activity status. In people with cognitive impairment, this willingness to exercise disappeared probably due to a decrease in self-care. Therefore, we advocate that elderly people with dental loss seek reasonable treatment as soon as possible, since it can improve their quality of life and the overall prognosis of systemic diseases (Papadaki and Anastassiadou, 2012; Gupta et al., 2019).

The loss of teeth has been associated with multiple systemic comorbidities, as the more teeth lost, the higher the death risk from all causes (Yu et al., 2021). Our study supports previous research that dentures can repair the loss of health due to tooth loss (Chen et al., 2020). In fact, socioeconomic status, such as education and income level, is highly correlated with access to dental care, as evidenced by the availability of dentures (Chalub et al., 2016; Elani et al., 2021). Older adults with impaired oral function can experience physical weakness and other adverse health outcomes, including death (Fried et al., 2001; Tanaka et al., 2018; Hakeem et al., 2021). Continued deterioration in oral health has been found to be a significant predictor of mortality (Tanaka et al., 2018; Gupta et al., 2019).

In addition, we also explored the effect of sex on the relationship between denture-wearing and moderate physical activity. It was found that denture-wearing and moderate physical activity were more likely to occur in older women with oral disease than in men. This may be due to differences in the physical structure, emotional characteristics, and social roles between men and women. However, after adjusting for confounding factors such as sex, age, and MMSE, moderate physical activity was still found to be associated with denture-wearing.

In summary, this study indirectly argues for a potential relationship between early treatment of oral disease and physical activity based on data from a large sample. We found that oral health is affected in humans following cognitive impairment. And our work may reveal specific changes in oral health that are influenced by cognitive state, which may be a critical connection in the interaction between the nervous system and the oral cavity. Therefore, the findings of this study may also have implications for the advancement of basic research on oral-neurovascular diseases. Previous research on cognitive impairment affecting oral health for overall health is extremely limited, and our study may provide new ideas to facilitate the study of cognitive effects on the oral health. 5,892 cases were included in this study, thereby reducing the possibility of too many variables and statistical analysis methods, which may lead to α error. We found an association between denture wearing and physical activity after controlling for covariates such as sociodemographic factors and the cognitive status. Poor oral hygiene may threaten the physical health of patients, and a moderate exercise program is positively associated with oral function (Farmer et al., 1988; Hakeem et al., 2019; Miyoshi et al., 2021; Mun et al., 2021). There are some limitations to this study. First, only denture wear data was included, since it was not possible to determine why patients did not wear dentures. It was perhaps because their oral cavity was in a healthy condition, or maybe they did not wear dentures for socioeconomic reasons despite having missing teeth. Therefore, the participants without dentures in this study is two types. First is healthy dentate who need not denture. The other is partially or fully edentulous, but not use denture. As the CHARLS database does not measure the number of teeth, future studies will need to distinguish between the two types of dental caries. A few confounding factors, such as cases with missing MMSE data, should also be considered when interpreting the study results. Patients with cognitive impairment may have been unable to complete the questionnaire, introducing potential selection bias. Additionally, due to the cross-sectional study design, we were unable to establish a causal relationship between wearing dentures and moderate physical activity. It is therefore necessary to conduct further research in the future on the potential causal mechanisms between poor oral health and physical activity. Also, many elderly people in developing countries may be less concerned about their general health in developing countries, where oral health is generally poor, so they may be reluctant to seek prosthetic restoration. There is a possibility that people who wear dentures are more motivated to exercise and are more focused on whole body and oral health. Therefore, the study may have screened for those who placed more importance on their health.

## Conclusion

We found a relationship between moderate physical activity and denture wearing in a population without cognitive impairment, even after controlling for age, sex, and cognitive impairment. A particular benefit of wearing dentures is that it delays or reduces mild cognitive impairment. The treatment of tooth loss in older adults should be sought as soon as possible in order to improve their quality of life and prevent frailty.

## Data availability statement

The data for this study were obtained from the CHARLS dataset, they can be found in the article/Supplementary material, further inquiries can be directed to the corresponding author.

## Ethics statement

Ethical review and approval were not required for the study on human participants in accordance with the local legislation and institutional requirements. Written informed consent was obtained from all participants for their participation in this study.

## Author contributions

YC: methodology, conceptualization, software, validation, formal analysis, data curation, and writing – original draft. ZL: data curation, methodology, and conceptualization. YS: conceptualization, methodology, and supervision. YZ: conceptualization, supervision, conceptualization, supervision, and methodology. ZH: formal analysis and data curation. XY: supervision and project administration. XK: data curation and writing – review and editing. JL: data curation and methodology. BQ: data curation and writing – review and editing. W-WL: supervision and project administration. HG: writing and validation. CG: supervision and methodology. KG: formal analysis and data curation. CS: software and validation. XL: writing – original draft, conceptualization, supervision, methodology, and funding acquisition. JC and SC: conceptualization, supervision, project administration, and funding acquisition. All authors contributed to the article and approved the submitted version.

## Footnotes

1
https://spssau.com/index.html

## References

[B1] Albani V. Nishio K. Ito T. Kotronia E. Moynihan P. Robinson L. (2021). Associations of poor oral health with frailty and physical functioning in the oldest old: Results from two studies in England and Japan. *BMC Geriatr.* 21:187. 10.1186/s12877-021-02081-5 33736595 PMC7977173

[B2] Bannwart L. C. de Moraes Melo Neto C. L. Goiato M. C. dos Santos D. M. da Silva Paiva C. A. de Araújo Moreno N. V. (2021). Oral Health-Related Quality of Life, Dry Mouth Sensation, and Level of Anxiety in Elderly Patients Rehabilitated with New Removable Dentures. *Eur. J. Dent.* [Epub ahead of print]. 10.1055/s-0041-1735796 34814220 PMC9339923

[B3] Castrejon-Perez R. C. Borges-Yañez S. A. (2012). association between the use of complete dentures and frailty in edentulous mexican elders. *J. Frailty Aging* 1 183–188. 10.14283/jfa.2012.28 27093319

[B4] Chalub L. L. F. H. Martins C. C. Ferreira R. C. Vargas A. M. D. (2016). Functional Dentition in Brazilian Adults: An Investigation of Social Determinants of Health (SDH) Using a Multilevel Approach. *PLoS One* 11:e0148859. 10.1371/journal.pone.0148859 26862892 PMC4749636

[B5] Chen X. Su D. Chen X. Chen Y. (2021). What intensity of exercise is most suitable for the elderly in China? A propensity score matching analysis. *BMC Public Health* 21:1396. 10.1186/s12889-021-11407-2 34261461 PMC8281566

[B6] Chen Y. Sun Y. Luo Z. Lin J. Qi B. Kang X. (2022). Potential Mechanism Underlying Exercise Upregulated Circulating Blood Exosome miR-215-5p to Prevent Necroptosis of Neuronal Cells and a Model for Early Diagnosis of Alzheimer’s Disease. *Front. Aging Neurosci.* 14:860364. 10.3389/fnagi.2022.860364 35615585 PMC9126031

[B7] Chen Y.-S. Cai Y.-X. Kang X.-R. Zhou Z.-H. Qi X. Ying C.-T. (2020). Predicting the risk of sarcopenia in elderly patients with patellar fracture: Development and assessment of a new predictive nomogram. *PeerJ.* 8:e8793. 10.7717/peerj.8793 32328345 PMC7166043

[B8] da Mata C. Allen P. F. McKenna G. J. Hayes M. Kashan A. (2019). The relationship between oral-health-related quality of life and general health in an elderly population: A cross-sectional study. *Gerodontology* 36 71–77. 10.1111/ger.12384 30536976

[B9] De la Rosa A. Olaso-Gonzalez G. Arc-Chagnaud C. Millan F. Salvador-Pascual A. García-Lucerga C. (2020). Physical exercise in the prevention and treatment of Alzheimer’s disease. *J. Sport Health Sci.* 9 394–404. 10.1016/j.jshs.2020.01.004 32780691 PMC7498620

[B10] Delwel S. Scherder E. J. A. Perez R. S. G. M. Hertogh C. M. P. M. Maier A. B. Lobbezoo F. (2018). Oral function of older people with mild cognitive impairment or dementia. *J. Oral. Rehabil.* 45 990–997. 10.1111/joor.12708 30126006

[B11] Dong B.-R. Gu X.-Q. Chen H.-Y. Gu J. Pan Z.-G. (2021). Development and Validation of a Nomogram to Predict Frailty Progression in Nonfrail Chinese Community-Living Older Adults. *J. Am Med. Dir. Assoc.* 22:2571-2578.e4. 10.1016/j.jamda.2021.05.020 34129830

[B12] Elani H. W. Batista A. F. M. Thomson W. M. Kawachi I. Chiavegatto Filho A. D. P. (2021). Predictors of tooth loss: A machine learning approach. *PLoS One* 16:e0252873. 10.1371/journal.pone.0252873 34143814 PMC8213149

[B13] Farmer M. E. Locke B. Z. Mościcki E. K. Dannenberg A. L. Larson D. B. Radloff L. S. (1988). physical activity and depressive symptoms: The nhanes i epidemiologic follow-up study. *Am. J. Epidemiol.* 128 1340–1351. 10.1093/oxfordjournals.aje.a115087 3264110

[B14] Farsai P. S. (2021). Cognitive Impairment in Older Adults and Oral Health Considerations. *Dent. Clin. North Am.* 65 345–360. 10.1016/j.cden.2020.11.008 33641757

[B15] Folstein M. F. Folstein S. E. McHugh P. R. (1975). Mini-mental state. *J. Psychiatric Res.* 12 189–198. 10.1016/0022-3956(75)90026-6 1202204

[B16] Fried L. P. Tangen C. M. Walston J. Newman A. B. Hirsch C. Gottdiener J. (2001). Frailty in Older Adults: Evidence for a Phenotype. *J. Gerontol. Series A* 56:M146-M157. 10.1093/gerona/56.3.M146 11253156

[B17] Garber C. E. Blissmer B. Deschenes M. R. Franklin B. A. Lamonte M. J. Lee I.-M. (2011). Quantity and Quality of Exercise for Developing and Maintaining Cardiorespiratory, Musculoskeletal, and Neuromotor Fitness in Apparently Healthy Adults: Guidance for Prescribing Exercise. *Med. Sci. Sports Exerc.* 43 1334–1359. 10.1249/MSS.0b013e318213fefb 21694556

[B18] Gill T. M. Williams C. S. Tinetti M. E. (1995). Assessing Risk for the Onset of Functional Dependence Among Older Adults: The Role of Physical Performance. *J.Am. Geriatric. Soc.* 43 603–609. 10.1111/j.1532-5415.1995.tb07192.x 7775716

[B19] Gondivkar S. M. Gadbail A. R. Gondivkar R. S. Sarode S. C. Sarode G. S. Patil S. (2019). Nutrition and oral health. *Disease-a-Month* 65 147–154. 10.1016/j.disamonth.2018.09.009 30293649

[B20] Gupta A. Felton D. A. Jemt T. Koka S. (2019). Rehabilitation of Edentulism and Mortality: A Systematic Review. *J. Prosthodontics* 28 526–535. 10.1111/jopr.12792 29573048

[B21] Hakeem F. F. Bernabé E. Sabbah W. (2019). Association between oral health and frailty: A systematic review of longitudinal studies. *Gerodontology* 36 205–215. 10.1111/ger.12406 31025772

[B22] Hakeem F. F. Bernabé E. Sabbah W. (2021). Association Between Oral Health and Frailty Among American Older Adults. *J. Am. Med. Dir. Assoc.* 22:559-563.e2. 10.1016/j.jamda.2020.07.023 32859517

[B23] Jiang S. Ding Y. Kang L. (2022). Impact of sarcopenia on intertrochanteric femoral fracture in the elderly. *PeerJ.* 10:e13445. 10.7717/peerj.13445 35726258 PMC9206433

[B24] Jin L. Lamster I. Greenspan J. Pitts N. Scully C. Warnakulasuriya S. (2016). Global burden of oral diseases: Emerging concepts, management and interplay with systemic health. *Oral. Dis.* 22 609–619. 10.1111/odi.12428 26704694

[B25] Jongsiriyanyong S. Limpawattana P. (2018). Mild Cognitive Impairment in Clinical Practice: A Review Article. *Am. J. Alzheimers Dis. Other Demen.* 33 500–507. 10.1177/1533317518791401 30068225 PMC10852498

[B26] Kim M.-S. Oh B. Yoo J. W. Han D.-H. (2020). The association between mastication and mild cognitive impairment in Korean adults. *Medicine* 99:e20653. 10.1097/MD.0000000000020653 32502052 PMC7306381

[B27] Lawton M. P. Brody E. M. (1969). Assessment of Older People: Self-Maintaining and Instrumental Activities of Daily Living. *Gerontologist* 9 179–186. 10.1093/geront/9.3_Part_1.179 5349366

[B28] Lee S. Sabbah W. (2018). Association between number of teeth, use of dentures and musculoskeletal frailty among older adults: Dental status and frailty. *Geriatr. Gerontol. Int.* 18 592–598. 10.1111/ggi.13220 29218839

[B29] López-Ortiz S. Valenzuela P. L. Seisdedos M. M. Morales J. S. Vega T. Castillo-García A. (2021). Exercise interventions in Alzheimer’s disease: A systematic review and meta-analysis of randomized controlled trials. *Ageing Res. Rev.* 72:101479. 10.1016/j.arr.2021.101479 34601135

[B30] Miyoshi S. Saito A. Shigeishi H. Ohta K. Sugiyama M. (2021). Relationship between oral and physical function and length of participation in long-term care prevention programs in community-dwelling older Japanese women. *Eur. Geriatr. Med.* 12 387–395. 10.1007/s41999-020-00424-w 33145741

[B31] Mo Y.-H. Su Y.-D. Dong X. Zhong J. Yang C. Deng W.-Y. (2022). Development and Validation of a Nomogram for Predicting Sarcopenia in Community-Dwelling Older Adults. *J.Am. Med. Dir. Assoc.* 23:715-721.e5. 10.1016/j.jamda.2021.11.023 34932988

[B32] Mun S. J. Jeon H. S. Choi E. S. Lee R. Kim S. H. Han S. Y. (2021). Oral health status of inpatients with varying physical activity limitations in rehabilitation wards: A cross-sectional study. *Medicine* 100:e26880. 10.1097/MD.0000000000026880 34397904 PMC8360428

[B33] Nazir M. A. AlGhamdi L. AlKadi M. AlBeajan N. AlRashoudi L. AlHussan M. (2018). The burden of Diabetes, Its Oral Complications and Their Prevention and Management. *Open Access Maced. J. Med. Sci.* 6 1545–1553. 10.3889/oamjms.2018.294 30159091 PMC6108795

[B34] Ogawa M. Satomi-Kobayashi S. Yoshida N. Tsuboi Y. Komaki K. Nanba N. (2021). Relationship between oral health and physical frailty in patients with cardiovascular disease. *J. Cardiol.* 77 131–138. 10.1016/j.jjcc.2020.07.016 32819801

[B35] Panza F. Lozupone M. Solfrizzi V. Sardone R. Dibello V. Di Lena L. (2018). Different Cognitive Frailty Models and Health- and Cognitive-related Outcomes in Older Age: From Epidemiology to Prevention. *JAD* 62 993–1012. 10.3233/JAD-170963 29562543 PMC5870024

[B36] Papadaki E. Anastassiadou V. (2012). Elderly complete denture wearers: A social approach to tooth loss: Tooth loss social outcomes in denture wearers. *Gerodontology* 29:e721-e727. 10.1111/j.1741-2358.2011.00550.x 21916954

[B37] Peres M. A. Macpherson L. M. D. Weyant R. J. Daly B. Venturelli R. Mathur M. R. (2019). Oral diseases: A global public health challenge. *Lancet* 394 249–260. 10.1016/S0140-6736(19)31146-8 31327369

[B38] Pflipsen M. Zenchenko Y. (2017). Nutrition for oral health and oral manifestations of poor nutrition and unhealthy habits. *Gen. Dent.* 65 36–43. 29099364

[B39] Poole D. Rossi C. Latronico N. Rossi G. Finazzi S. Bertolini G. (2012). Comparison between SAPS II and SAPS 3 in predicting hospital mortality in a cohort of 103 Italian ICUs. Is new always better? *Intensive Care Med.* 38 1280–1288. 10.1007/s00134-012-2578-0 22584793

[B40] Preshaw P. M. Walls A. W. G. Jakubovics N. S. Moynihan P. J. Jepson N. J. A. Loewy Z. (2011). Association of removable partial denture use with oral and systemic health. *J. Dent.* 39 711–719. 10.1016/j.jdent.2011.08.018 21924317

[B41] Quine S. Morrell S. (2009). Hopelessness, depression and oral health concerns reported by community dwelling older Australians. *Community Dent. Health* 26 177–182. 19780359

[B42] Sabbah W. Slade G. D. Sanders A. E. Bernabé E. (2020). Denture wearing and mortality risk in edentulous American adults: A propensity score analysis. *J. Dent.* 100:103360. 10.1016/j.jdent.2020.103360 32404256

[B43] Sanchez G. F. L. Smith L. Koyanagi A. Grabovac I. Yang L. Veronese N. (2020). Associations between self-reported physical activity and oral health: A cross-sectional analysis in 17,777 Spanish adults. *Br. Dent. J.* 228 361–365. 10.1038/s41415-020-1306-3 32170257

[B44] Shi Q. Yan X. Wang J. Zhang X. (2021). Collagen Family Genes Associated with Risk of Recurrence after Radiation Therapy for Vestibular Schwannoma and Pan-Cancer Analysis. *Dis. Markers* 2021:7897994. 10.1155/2021/7897994 34691289 PMC8528601

[B45] Strauss J. Lei X. Park A. Shen Y. Smith J. P. Yang Z. (2010). Health Outcomes and Socio-economic Status Among the Elderly in China: Evidence from the CHARLS Pilot. *Popul. Ageing* 3 111–142. 10.1007/s12062-011-9033-9 23539329 PMC3608278

[B46] Tahami Monfared A. A. Byrnes M. J. White L. A. Zhang Q. (2022). Alzheimer’s Disease: Epidemiology and Clinical Progression. *Neurol. Ther.* 11 553–569. 10.1007/s40120-022-00338-8 35286590 PMC9095793

[B47] Tanaka T. Takahashi K. Hirano H. Kikutani T. Watanabe Y. Ohara Y. (2018). Oral Frailty as a Risk Factor for Physical Frailty and Mortality in Community-Dwelling Elderly. *J. Gerontol.* 73 1661–1667. 10.1093/gerona/glx225 29161342

[B48] Watanabe Y. Okada K. Kondo M. Matsushita T. Nakazawa S. Yamazaki Y. (2020). Oral health for achieving longevity. *Geriatr. Gerontol. Int.* 20 526–538. 10.1111/ggi.13921 32307825

[B49] Ying C. Guo C. Wang Z. Chen Y. Sun J. Qi X. (2021). Prediction Modeling Based on the Hospital for Special Surgery (HSS) Knee Score for Poor Postoperative Functional Prognosis of Elderly Patients with Patellar Fractures. *Biomed. Res. Int.* 2021:6620504. 10.1155/2021/6620504 34912895 PMC8668305

[B50] Yu Y.-H. Cheung W. S. Steffensen B. Miller D. R. (2021). Number of teeth is associated with all-cause and disease-specific mortality. *BMC Oral. Health* 21:568. 10.1186/s12903-021-01934-0 34749715 PMC8574051

[B51] Yun J. Ki S. Kim J. Chon D. Shin S. Lee Y. (2020). Relationships between cognitive function and frailty in older Korean adults: The moderating effect of the number of teeth. *Arch. Gerontol. Geriatr.* 91:104213. 10.1016/j.archger.2020.104213 32805701

[B52] Zhang L. Cui H. Chen Q. Li Y. Yang C. Yang Y. (2021). A web-based dynamic Nomogram for predicting instrumental activities of daily living disability in older adults: A nationally representative survey in China. *BMC Geriatr.* 21:311. 10.1186/s12877-021-02223-9 34001030 PMC8127258

[B53] Zhao X. Zhou Y. Wei K. Bai X. Zhang J. Zhou M. (2021). Associations of sensory impairment and cognitive function in middle-aged and older Chinese population: The China Health and Retirement Longitudinal Study. *J. Glob. Health* 11:08008. 10.7189/jogh.11.08008 34956639 PMC8684796

[B54] Zhong H. (2011). Effect of patient reimbursement method on health-care utilization: Evidence from China. *Health Econ.* 20 1312–1329. 10.1002/hec.1670 20882574

[B55] Zhou Y. Hu Y. Luo J. Li Y. Liu H. Sun X. (2022). Association Between Sensory Loss and Falls Among Middle-Aged and Older Chinese Population: Cross-Sectional and Longitudinal Analyses. *Front. Med.* 8:810159. 10.3389/fmed.2021.810159 35096898 PMC8793905

