# Supplementary material for: The effect of denture-wearing on physical activity is associated with cognitive impairment in the elderly: A cross-sectional study based on the CHARLS database
Source: Front Neurosci. 2022 Aug 16;16:925398. doi: 10.3389/fnins.2022.925398 (PMC9425833; doi:10.3389/fnins.2022.925398)
Supplement: Supplementary file 1 [file Table_1.docx]

| Supplementary Table 1. Study participants' characteristics according to denture wear status. | | | | | | | | | |
| --- | --- | --- | --- | --- | --- | --- | --- | --- | --- |
| Characteristics | No cognitive impairment | | | |  | Cognitive impairment | | | |
|  | Wear_dentures(%) | | Total | *P* value |  | Wear_dentures(%) | | Total | *P* value |
|  | No(n=2426) | Yes(n=1657) |  |  |  | No(n=1185) | Yes(n=624) |  |  |
| Age, scores (Mean±SE) | 67.01±5.56 | 67.88±5.74 | 67.36±5.65 | <0.001** |  | 67.84±6.45 | 68.38±6.37 | 68.02±6.43 | 0.091 |
| Sex, n (%) |  |  |  |  |  |  |  |  |  |
| Female | 939(38.71) | 778(46.95) | 1717(42.05) | <0.001** |  | 630(53.16) | 383(61.38) | 1013(56.00) | 0.001** |
| Male | 1487(61.29) | 879(53.05) | 2366(57.95) |  |  | 555(46.84) | 241(38.62) | 796(44.00) |  |
| Residence, n (%) |  |  |  |  |  |  |  |  |  |
| Urban | 817(33.82) | 657(40.01) | 1474(36.32) | <0.001** |  | 185(15.69) | 117(18.87) | 302(16.79) | 0.086 |
| Rural areas | 1599(66.18) | 985(59.99) | 2584(63.68) |  |  | 994(84.31) | 503(81.13) | 1497(83.21) |  |
| Education, n (%) |  |  |  |  |  |  |  |  |  |
| Elementary | 1189(49.01) | 799(48.22) | 1988(48.69) | 0.05 |  | 481(40.59) | 227(36.38) | 708(39.14) | 0.139 |
| Illiteracy | 381(15.70) | 223(13.46) | 604(14.79) |  |  | 423(35.70) | 227(36.38) | 650(35.93) |  |
| Secondary | 856(35.28) | 635(38.32) | 1491(36.52) |  |  | 281(23.71) | 170(27.24) | 451(24.93) |  |
| Marital status, n (%) |  |  |  |  |  |  |  |  |  |
| Divorced or Separated | 33(1.36) | 23(1.39) | 56(1.37) | 0.011* |  | 10(0.84) | 12(1.92) | 22(1.22) | 0.088 |
| Married | 2112(87.06) | 1392(84.01) | 3504(85.82) |  |  | 935(78.90) | 476(76.28) | 1411(78.00) |  |
| Never married | 12(0.49) | 4(0.24) | 16(0.39) |  |  | 12(1.01) | 3(0.48) | 15(0.83) |  |
| Widowed | 269(11.09) | 238(14.36) | 507(12.42) |  |  | 228(19.24) | 133(21.31) | 361(19.96) |  |
| Smoking, n (%) |  |  |  |  |  |  |  |  |  |
| Never | 1161(90.70) | 845(90.96) | 2006(90.81) | 0.703 |  | 639(92.74) | 386(92.79) | 1025(92.76) | 0.957 |
| Quit | 65(5.08) | 41(4.41) | 106(4.80) |  |  | 30(4.35) | 19(4.57) | 49(4.43) |  |
| Smoking | 54(4.22) | 43(4.63) | 97(4.39) |  |  | 20(2.90) | 11(2.64) | 31(2.81) |  |
| Drinking, n (%) |  |  |  |  |  |  |  |  |  |
| Drink but less than once a month | 197(8.12) | 160(9.66) | 357(8.74) | 0.232 |  | 60(5.06) | 46(7.37) | 106(5.86) | 0.139 |
| Drink more than once a month | 743(30.63) | 496(29.93) | 1239(30.35) |  |  | 272(22.95) | 139(22.28) | 411(22.72) |  |
| No | 1486(61.25) | 1001(60.41) | 2487(60.91) |  |  | 853(71.98) | 439(70.35) | 1292(71.42) |  |
| Social activity, n (%) |  |  |  |  |  |  |  |  |  |
| No | 1114(45.92) | 639(38.56) | 1753(42.93) | <0.001** |  | 671(56.62) | 334(53.53) | 1005(55.56) | 0.207 |
| Yes | 1312(54.08) | 1018(61.44) | 2330(57.07) |  |  | 514(43.38) | 290(46.47) | 804(44.44) |  |
| Self-report health conditions, n (%) |  |  |  |  |  |  |  |  | 0.098 |
| Very good | 274(11.29) | 172(10.38) | 446(10.92) |  |  | 122(10.30) | 59(9.46) | 181(10.01) |  |
| Good | 297(12.24) | 202(12.19) | 499(12.22) |  |  | 122(10.30) | 57(9.13) | 179(9.90) |  |
| Fair | 1218(50.21) | 880(53.11) | 2098(51.38) | 0.42 |  | 532(44.93) | 319(51.12) | 851(47.07) |  |
| Poor | 506(20.86) | 323(19.49) | 829(20.30) |  |  | 317(26.77) | 138(22.12) | 455(25.17) |  |
| Very poor | 131(5.40) | 80(4.83) | 211(5.17) |  |  | 91(7.69) | 51(8.17) | 142(7.85) |  |
| Hypertension, n (%) |  |  |  |  |  |  |  |  |  |
| No | 1317(82.62) | 910(83.11) | 2227(82.82) | 0.744 |  | 655(81.88) | 341(79.49) | 996(81.04) | 0.309 |
| Yes | 277(17.38) | 185(16.89) | 462(17.18) |  |  | 145(18.13) | 88(20.51) | 233(18.96) |  |
| Dyslipidemia, n (%) |  |  |  |  |  |  |  |  |  |
| No | 1737(86.68) | 1144(86.02) | 2881(86.41) | 0.585 |  | 941(89.79) | 464(84.83) | 1405(88.09) | 0.004** |
| Yes | 267(13.32) | 186(13.98) | 453(13.59) |  |  | 107(10.21) | 83(15.17) | 190(11.91) |  |
| Diabetes, n (%) |  |  |  |  |  |  |  |  |  |
| No | 2040(94.18) | 1358(92.95) | 3398(93.69) | 0.134 |  | 1028(93.88) | 527(93.61) | 1555(93.79) | 0.826 |
| Yes | 126(5.82) | 103(7.05) | 229(6.31) |  |  | 67(6.12) | 36(6.39) | 103(6.21) |  |
| Chronic lung diseases, n (%) |  |  |  |  |  |  |  |  |  |
| No | 2016(93.68) | 1379(93.36) | 3395(93.55) | 0.704 |  | 956(93.63) | 515(93.30) | 1471(93.52) | 0.796 |
| Yes | 136(6.32) | 98(6.64) | 234(6.45) |  |  | 65(6.37) | 37(6.70) | 102(6.48) |  |
| History of heart attack, n (%) |  |  |  |  |  |  |  |  |  |
| No | 1818(90.40) | 1182(89.07) | 3000(89.87) | 0.213 |  | 940(92.43) | 455(87.84) | 1395(90.88) | 0.003** |
| Yes | 193(9.60) | 145(10.93) | 338(10.13) |  |  | 77(7.57) | 63(12.16) | 140(9.12) |  |
| History of Stroke, n (%) |  |  |  |  |  |  |  |  |  |
| No | 2232(94.22) | 1510(93.56) | 3742(93.95) | 0.391 |  | 1086(93.94) | 563(92.45) | 1649(93.43) | 0.227 |
| Yes | 137(5.78) | 104(6.44) | 241(6.05) |  |  | 70(6.06) | 46(7.55) | 116(6.57) |  |
| Kidney disease, n (%) |  |  |  |  |  |  |  |  |  |
| No | 2118(95.45) | 1459(95.48) | 3577(95.46) | 0.959 |  | 1047(95.18) | 557(95.70) | 1604(95.36) | 0.628 |
| Yes | 101(4.55) | 69(4.52) | 170(4.54) |  |  | 53(4.82) | 25(4.30) | 78(4.64) |  |
| Memory related disease, n (%) |  |  |  |  |  |  |  |  |  |
| No | 2346(98.28) | 1591(97.91) | 3937(98.13) | 0.39 |  | 1116(96.79) | 593(96.90) | 1709(96.83) | 0.905 |
| Yes | 41(1.72) | 34(2.09) | 75(1.87) |  |  | 37(3.21) | 19(3.10) | 56(3.17) |  |
| Brain damage, n (%) |  |  |  |  |  |  |  |  |  |
| No | 2258(97.83) | 1513(96.99) | 3771(97.49) | 0.099 |  | 1057(96.44) | 553(94.37) | 1610(95.72) | 0.045* |
| Yes | 50(2.17) | 47(3.01) | 97(2.51) |  |  | 39(3.56) | 33(5.63) | 72(4.28) |  |
| Emotiol nervous or psychiatric problems, n (%) |  |  |  |  |  |  |  |  |  |
| No | 2374(99.04) | 1623(99.02) | 3997(99.03) | 0.958 |  | 1163(99.15) | 601(98.20) | 1764(98.82) | 0.079 |
| Yes | 23(0.96) | 16(0.98) | 39(0.97) |  |  | 10(0.85) | 11(1.80) | 21(1.18) |  |
| Cancer or malignant tumour, n (%) |  |  |  |  |  |  |  |  |  |
| No | 2367(98.42) | 1614(98.41) | 3981(98.42) | 0.989 |  | 1163(99.06) | 614(98.87) | 1777(99.00) | 0.7 |
| Yes | 38(1.58) | 26(1.59) | 64(1.58) |  |  | 11(0.94) | 7(1.13) | 18(1.00) |  |
| Asthma, n (%) |  |  |  |  |  |  |  |  |  |
| No | 2277(97.89) | 1534(97.09) | 3811(97.57) | 0.109 |  | 1077(96.94) | 580(96.83) | 1657(96.90) | 0.899 |
| Yes | 49(2.11) | 46(2.91) | 95(2.43) |  |  | 34(3.06) | 19(3.17) | 53(3.10) |  |
| Arthritis or rheumatism, n (%) |  |  |  |  |  |  |  |  |  |
| No | 1426(89.18) | 1005(89.10) | 2431(89.15) | 0.944 |  | 629(88.97) | 334(86.30) | 963(88.03) | 0.195 |
| Yes | 173(10.82) | 123(10.90) | 296(10.85) |  |  | 78(11.03) | 53(13.70) | 131(11.97) |  |
| Liver disease, n (%) |  |  |  |  |  |  |  |  |  |
| No | 2222(95.94) | 1515(95.40) | 3737(95.72) | 0.414 |  | 1123(97.91) | 573(96.95) | 1696(97.58) | 0.22 |
| Yes | 94(4.06) | 73(4.60) | 167(4.28) |  |  | 24(2.09) | 18(3.05) | 42(2.42) |  |
| Stomach or other digestive diseases, n (%) |  |  |  |  |  |  |  |  |  |
| No | 1704(89.59) | 1156(90.45) | 2860(89.94) | 0.427 |  | 781(90.08) | 425(91.01) | 1206(90.40) | 0.584 |
| Yes | 198(10.41) | 122(9.55) | 320(10.06) |  |  | 86(9.92) | 42(8.99) | 128(9.60) |  |
| Physical disabilities, n (%) |  |  |  |  |  |  |  |  |  |
| No | 2199(96.91) | 1525(98.01) | 3724(97.36) | 0.038* |  | 1042(95.86) | 560(96.55) | 1602(96.10) | 0.487 |
| Yes | 70(3.09) | 31(1.99) | 101(2.64) |  |  | 45(4.14) | 20(3.45) | 65(3.90) |  |
| Vision problem, n (%) |  |  |  |  |  |  |  |  |  |
| No | 2106(96.21) | 1447(96.15) | 3553(96.18) | 0.923 |  | 948(94.05) | 511(94.63) | 1459(94.25) | 0.639 |
| Yes | 83(3.79) | 58(3.85) | 141(3.82) |  |  | 60(5.95) | 29(5.37) | 89(5.75) |  |
| Hearing problem, n (%) |  |  |  |  |  |  |  |  |  |
| No | 1961(94.19) | 1337(93.69) | 3298(93.99) | 0.544 |  | 884(92.08) | 459(92.91) | 1343(92.37) | 0.572 |
| Yes | 121(5.81) | 90(6.31) | 211(6.01) |  |  | 76(7.92) | 35(7.09) | 111(7.63) |  |
| Speech impediment, n (%) |  |  |  |  |  |  |  |  |  |
| No | 2409(99.71) | 1648(99.82) | 4057(99.75) | 0.495 |  | 1167(99.40) | 612(99.35) | 1779(99.39) | 0.891 |
| Yes | 7(0.29) | 3(0.18) | 10(0.25) |  |  | 7(0.60) | 4(0.65) | 11(0.61) |  |
| Fallen last two years, n (%) |  |  |  |  |  |  |  |  |  |
| No | 1964(80.96) | 1338(80.75) | 3302(80.87) | 0.868 |  | 923(77.89) | 483(77.40) | 1406(77.72) | 0.813 |
| Yes | 462(19.04) | 319(19.25) | 781(19.13) |  |  | 262(22.11) | 141(22.60) | 403(22.28) |  |
| Physical activities, n (%) |  |  |  |  |  |  |  |  |  |
| Mild physical activity | 1126(46.41) | 746(45.02) | 1872(45.85) | 0.004** |  | 574(48.44) | 302(48.40) | 876(48.42) | 0.293 |
| Moderate physical activity | 789(32.52) | 613(36.99) | 1402(34.34) |  |  | 301(25.40) | 176(28.21) | 477(26.37) |  |
| Vigorous physical activity | 511(21.06) | 298(17.98) | 809(19.81) |  |  | 310(26.16) | 146(23.40) | 456(25.21) |  |
| ADL, scores (Mean±SE) | 4.64±3.10 | 4.72±3.03 | 4.68±3.07 | 0.471 |  | 5.51±3.32 | 5.23±3.25 | 5.41±3.29 | 0.171 |
| IADL, scores (Mean±SE) | 6.66±1.79 | 6.59±1.79 | 6.63±1.79 | 0.298 |  | 7.80±3.29 | 7.46±2.86 | 7.68±3.15 | 0.08 |
| MMSE, scores (Mean±SE) | 24.56±3.17 | 24.86±3.09 | 24.68±3.14 | 0.002** |  | 15.74±4.33 | 16.22±4.28 | 15.9±4.32 | 0.024* |
| *P* value obtained by t test and Chi-Squared test. * : P ＜ 0. 05; ** : P ＜ 0. 01; *** : P ＜ 0. 001。. ADL = activities of daily living , IADL= Instrumental Activities of Daily Living, MMSE = The Mini-Mental State Examination, SE = standard error. | | | | | | | | | |
|  |  |  |  |  |  |  |  |  |  |
